# 2-(4-Fluoro­benzyl­idene)propane­dinitrile: monoclinic polymorph

**DOI:** 10.1107/S1600536813006235

**Published:** 2013-03-09

**Authors:** Ahmed M. El-Agrody, Mohamed A. Al-Omar, Abd El-Galil E. Amr, Seik Weng Ng, Edward R. T. Tiekink

**Affiliations:** a Chemistry Department, Faculty of Science, King Khalid University, Abha 61413, PO Box 9004, Saudi Arabia; b Chemistry Department, Faculty of Science, Al-Azhar University, Nasr City, Cairo 11884, Egypt; c Pharmaceutical Chemistry Department, College of Pharmacy, King Saud University, Riyadh 11451, Saudi Arabia; d Drug Exploration & Development Chair (DEDC), College of Pharmacy, King Saud University, Riyadh 11451, Saudi Arabia; e Applied Organic Chemistry Department, National Research Center, Dokki 12622, Cairo, Egypt; f Department of Chemistry, University of Malaya, 50603 Kuala Lumpur, Malaysia; g Chemistry Department, Faculty of Science, King Abdulaziz University, PO Box 80203 Jeddah, Saudi Arabia

## Abstract

The title compound, C_10_H_5_FN_2_, is a monoclinic (*P*2_1_/*c*) polymorph of the previously reported triclinic (*P*-1) form [Anti­pin *et al.* (2003 ▶). *J. Mol. Struct*. **650**, 1–20]. The 13 non-H atoms in the title polymorph are almost coplanar (r.m.s. deviation = 0.020 Å); a small twist between the fluoro­benzene and dinitrile groups [C—C—C—C torsion angle = 175.49 (16)°] is evident in the triclinic polymorph. In the crystal, C—H⋯N inter­actions lead to supra­molecular layers parallel to (-101); these are connected by C—F⋯π inter­actions.

## Related literature

For background to the chemistry and biological activity of 4*H*-pyran derivatives, see: El-Agrody *et al.* (2011 ▶); Sabry *et al.* (2011 ▶). For the structure of the triclinic polymorph, see: Anti­pin *et al.* (2003 ▶); Ng & Tiekink (2013 ▶).
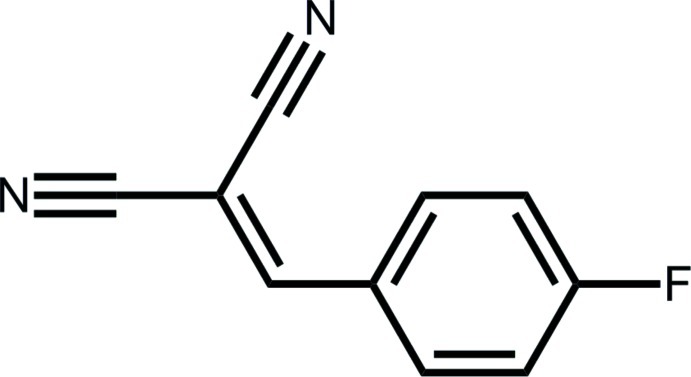


## Experimental

### 

#### Crystal data

C_10_H_5_FN_2_
*M* *_r_* = 172.16
Monoclinic, 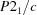

*a* = 9.1491 (9) Å
*b* = 12.7961 (14) Å
*c* = 7.5828 (11) Å
β = 106.317 (13)°
*V* = 851.98 (18) Å^3^
*Z* = 4
Mo *K*α radiation
μ = 0.10 mm^−1^
*T* = 295 K
0.35 × 0.15 × 0.05 mm

#### Data collection

Agilent SuperNova Dual diffractometer with an Atlas detector
Absorption correction: multi-scan (*CrysAlis PRO*; Agilent, 2011 ▶) *T*_min_ = 0.892, *T*_max_ = 1.000
7732 measured reflections
1957 independent reflections
1149 reflections with *I* > 2σ(*I*)
*R*_int_ = 0.045

#### Refinement

*R*[*F* ^2^ > 2σ(*F* ^2^)] = 0.060
*wR*(*F* ^2^) = 0.190
*S* = 1.09
1957 reflections
118 parameters
H-atom parameters constrained
Δρ_max_ = 0.15 e Å^−3^
Δρ_min_ = −0.18 e Å^−3^

### 

Data collection: *CrysAlis PRO* (Agilent, 2011 ▶); cell refinement: *CrysAlis PRO*; data reduction: *CrysAlis PRO*; program(s) used to solve structure: *SHELXS97* (Sheldrick, 2008 ▶); program(s) used to refine structure: *SHELXL97* (Sheldrick, 2008 ▶); molecular graphics: *ORTEP-3 for Windows* (Farrugia, 2012 ▶), *QMol* (Gans & Shalloway, 2001 ▶) and *DIAMOND* (Brandenburg, 2006 ▶); software used to prepare material for publication: *publCIF* (Westrip, 2010 ▶).

## Supplementary Material

Crystal structure: contains datablock(s) global, I. DOI: 10.1107/S1600536813006235/hb7051sup1.cif

Structure factors: contains datablock(s) I. DOI: 10.1107/S1600536813006235/hb7051Isup2.hkl

Supplementary material file. DOI: 10.1107/S1600536813006235/hb7051Isup3.cml

Additional supplementary materials:  crystallographic information; 3D view; checkCIF report

## Figures and Tables

**Table 1:** Hydrogen-bond geometry (Å, °) *Cg*1 is the centroid of the C1–C6 ring.

*D*—H⋯*A*	*D*—H	H⋯*A*	*D*⋯*A*	*D*—H⋯*A*
C3—H3⋯N1^i^	0.93	2.61	3.478 (4)	155
C7—H7⋯N2^ii^	0.93	2.51	3.424 (3)	167
C4—F1⋯*Cg*1^iii^	1.35 (1)	3.59 (1)	3.573 (2)	79 (1)

Symmetry codes: (i) 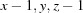; (ii) 
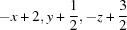
; (iii) 
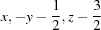
.

**Fig. 1.**
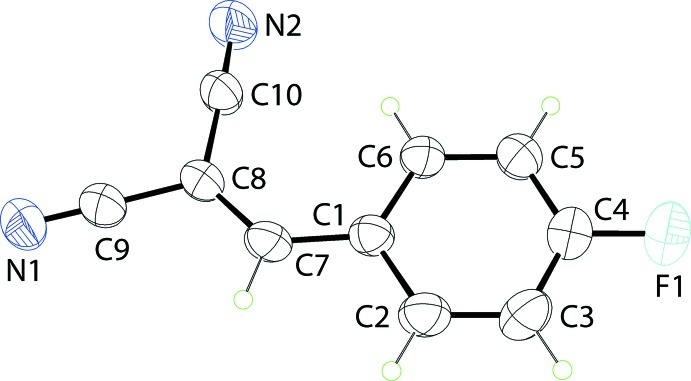
The molecular structures of (I) showing displacement ellipsoids at the 35% probability level.

**Fig. 2.**
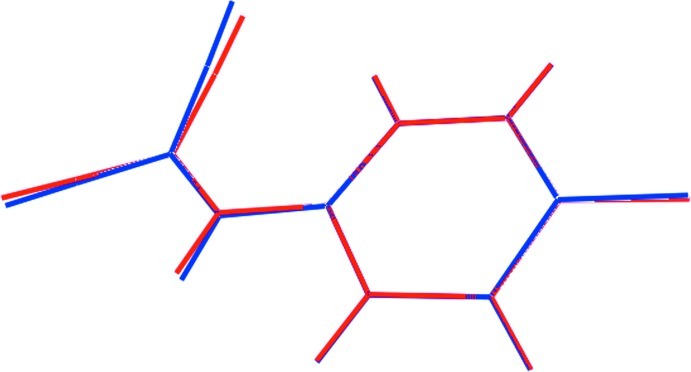
Overlay diagram of (I) (red) with the triclinic form (blue). The molecules are overlaid so that the benzene rings are superimposed.

**Fig. 3.**
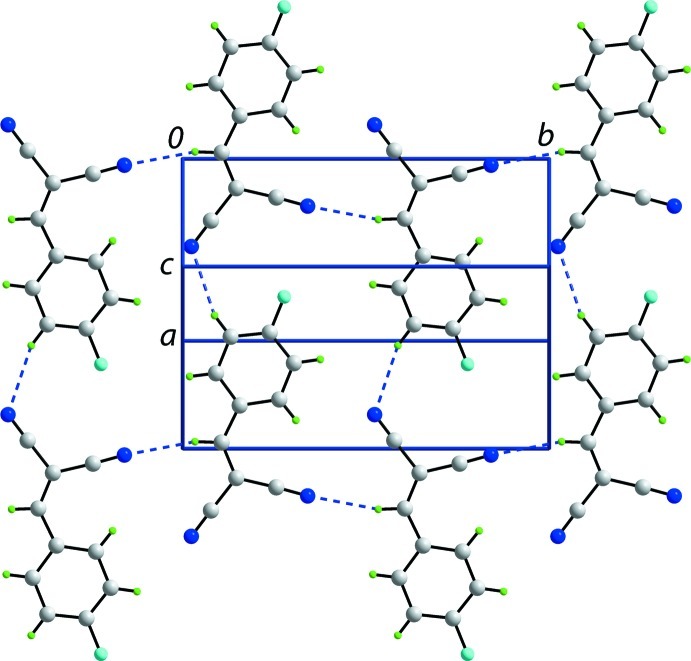
A view of the supramolecular layer parallel to (1 0 1) in (I) sustained by C—H···N interactions, shown as blue dashed lines.

**Fig. 4.**
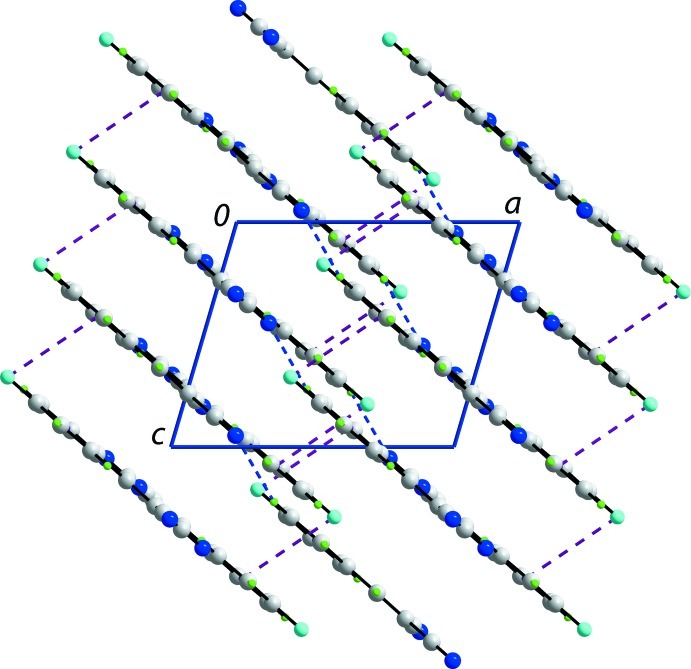
A view in projection down the b axis of the crystal packing in (I). The C—H···N and C—F···π interactions are shown as blue and purple dashed lines, respectively.

## supplementary crystallographic information

### Comment

In continuation of a program on the chemistry of 4*H*-pyran derivatives (El-Agrody *et al.*, 2011; Sabry *et al.*, 2011), the title compound was isolated from a failed reaction, see Experimental, as a monoclinic polymorph (*P*2_1_/*c*) of the previously reported triclinic (*P*1) form (Antipin *et al.*, 2003). In fact, both forms were characterized from the same reaction product (Ng & Tiekink, 2013).

In (I), Fig. 1, the 13 non-hydrogen atoms are almost co-planar with a r.m.s. deviation of 0.020 Å, and with maximum deviations of 0.028 (2) Å for the C5 atom and -0.028 (2) Å for C2. This contrasts the small twist found in the triclinic form (r.m.s. deviation = 0.062 Å) as seen in the C2—C1—C7—C8 torsion angle of 175.49 (16)° (Ng & Tiekink, 2013), which compares to -180.0 (2)° in (I); this difference is emphasized in the overlay diagram shown in Fig. 2.

In the crystal, supramolecular layers mediated by C—H···N interactions are formed parallel to (1 0 1), Fig. 2 and Table 1, and these are connected into a three-dimensional array by C—F···π contacts, Fig. 4 and Table 1. This pattern of interactions is in stark contrast to that in the triclinic polymorph whereby C—H···N interactions, involving one N atom only, lead to supramolecular chains which are connected into double chains by weak π—π contacts (Ng & Tiekink, 2013).

### Experimental

A solution of 6-bromo-1-naphthol (0.01 mol) in EtOH (30 ml) was treated with 4-fluoro-1-(2,2-dicyanovinyl)benzene (0.01 mol) and piperidine (0.5 ml). The reaction mixture was heated until complete precipitation occurred (reaction time: 60 min). The solid product which formed was collected by filtration and recrystallized from ethanol to give the title compound, *i.e*. unreacted 4-fluoro-1-(2,2-dicyanovinyl)benzene, as both triclinic (Ng & Tiekink, 2013) and monoclinic (I) polymorphs. Both crystal forms have the appearance of yellow prisms.

### Refinement

The C-bound H atoms were geometrically placed (C–H = 0.93 Å) and refined as riding with *U_iso_*(H) = 1.2*U_eq_*(C).

### Crystal data

**Table:**

C_10_H_5_FN_2_	*F*(000) = 352
*M**_r_* = 172.16	*D*_x_ = 1.342 Mg m^−^^3^
Monoclinic, *P*2_1_/*c*	Mo *K*α radiation, λ = 0.71073 Å
Hall symbol: -P 2ybc	Cell parameters from 1835 reflections
*a* = 9.1491 (9) Å	θ = 3.1–27.5°
*b* = 12.7961 (14) Å	µ = 0.10 mm^−^^1^
*c* = 7.5828 (11) Å	*T* = 295 K
β = 106.317 (13)°	Prism, yellow
*V* = 851.98 (18) Å^3^	0.35 × 0.15 × 0.05 mm
*Z* = 4	

### Data collection

**Table:**

Agilent SuperNova Dual diffractometer with an Atlas detector	1957 independent reflections
Radiation source: SuperNova (Mo) X-ray Source	1149 reflections with *I* > 2σ(*I*)
Mirror monochromator	*R*_int_ = 0.045
Detector resolution: 10.4041 pixels mm^-1^	θ_max_ = 27.6°, θ_min_ = 3.2°
ω scan	*h* = −11→11
Absorption correction: multi-scan (*CrysAlis PRO*; Agilent, 2011)	*k* = −16→14
*T*_min_ = 0.892, *T*_max_ = 1.000	*l* = −9→9
7732 measured reflections	

### Refinement

**Table:**

Refinement on *F*^2^	Primary atom site location: structure-invariant direct methods
Least-squares matrix: full	Secondary atom site location: difference Fourier map
*R*[*F*^2^ > 2σ(*F*^2^)] = 0.060	Hydrogen site location: inferred from neighbouring sites
*wR*(*F*^2^) = 0.190	H-atom parameters constrained
*S* = 1.09	*w* = 1/[σ^2^(*F*_o_^2^) + (0.0742*P*)^2^ + 0.0917*P*] where *P* = (*F*_o_^2^ + 2*F*_c_^2^)/3
1957 reflections	(Δ/σ)_max_ < 0.001
118 parameters	Δρ_max_ = 0.15 e Å^−^^3^
0 restraints	Δρ_min_ = −0.18 e Å^−^^3^

### Special details

**Table:**

Geometry. All e.s.d.'s (except the e.s.d. in the dihedral angle between two l.s. planes) are estimated using the full covariance matrix. The cell e.s.d.'s are taken into account individually in the estimation of e.s.d.'s in distances, angles and torsion angles; correlations between e.s.d.'s in cell parameters are only used when they are defined by crystal symmetry. An approximate (isotropic) treatment of cell e.s.d.'s is used for estimating e.s.d.'s involving l.s. planes.
Refinement. Refinement of *F*^2^ against ALL reflections. The weighted *R*-factor *wR* and goodness of fit *S* are based on *F*^2^, conventional *R*-factors *R* are based on *F*, with *F* set to zero for negative *F*^2^. The threshold expression of *F*^2^ > σ(*F*^2^) is used only for calculating *R*-factors(gt) *etc*. and is not relevant to the choice of reflections for refinement. *R*-factors based on *F*^2^ are statistically about twice as large as those based on *F*, and *R*- factors based on ALL data will be even larger.

### Fractional atomic coordinates and isotropic or equivalent isotropic displacement parameters (Å^2^)

**Table:**

	*x*	*y*	*z*	*U*_iso_*/*U*_eq_	
F1	0.35050 (15)	0.22275 (14)	0.1895 (2)	0.0959 (6)	
N1	1.2165 (3)	0.47627 (19)	0.9490 (4)	0.1097 (9)	
N2	1.0706 (2)	0.15887 (19)	0.8205 (3)	0.0900 (8)	
C1	0.7452 (2)	0.34429 (17)	0.5304 (3)	0.0566 (6)	
C2	0.6299 (3)	0.4089 (2)	0.4263 (3)	0.0704 (7)	
H2	0.6434	0.4809	0.4340	0.084*	
C3	0.4968 (3)	0.3689 (2)	0.3126 (3)	0.0775 (7)	
H3	0.4209	0.4129	0.2442	0.093*	
C4	0.4797 (2)	0.2624 (2)	0.3035 (3)	0.0685 (7)	
C5	0.5886 (2)	0.1964 (2)	0.4027 (3)	0.0651 (6)	
H5	0.5733	0.1245	0.3937	0.078*	
C6	0.7208 (2)	0.23631 (18)	0.5158 (3)	0.0606 (6)	
H6	0.7952	0.1911	0.5836	0.073*	
C7	0.8788 (2)	0.39364 (18)	0.6487 (3)	0.0627 (6)	
H7	0.8763	0.4663	0.6451	0.075*	
C8	1.0061 (2)	0.35367 (18)	0.7630 (3)	0.0611 (6)	
C9	1.1232 (3)	0.4227 (2)	0.8665 (3)	0.0773 (7)	
C10	1.0398 (2)	0.2445 (2)	0.7927 (3)	0.0648 (6)	

### Atomic displacement parameters (Å^2^)

**Table:**

	*U*^11^	*U*^22^	*U*^33^	*U*^12^	*U*^13^	*U*^23^
F1	0.0668 (9)	0.1132 (15)	0.0951 (11)	−0.0038 (8)	0.0019 (8)	−0.0117 (9)
N1	0.1033 (16)	0.0584 (16)	0.131 (2)	−0.0124 (13)	−0.0271 (14)	0.0014 (13)
N2	0.0849 (14)	0.0549 (15)	0.1148 (19)	0.0018 (11)	0.0028 (12)	0.0019 (12)
C1	0.0644 (12)	0.0469 (14)	0.0582 (12)	0.0029 (9)	0.0169 (9)	0.0002 (9)
C2	0.0809 (15)	0.0528 (15)	0.0747 (14)	0.0089 (11)	0.0174 (12)	0.0037 (11)
C3	0.0714 (14)	0.080 (2)	0.0734 (15)	0.0198 (13)	0.0088 (11)	0.0048 (13)
C4	0.0595 (13)	0.080 (2)	0.0642 (14)	−0.0006 (11)	0.0139 (10)	−0.0066 (11)
C5	0.0686 (13)	0.0576 (15)	0.0684 (13)	−0.0032 (11)	0.0179 (11)	−0.0039 (10)
C6	0.0639 (12)	0.0516 (14)	0.0632 (12)	0.0033 (10)	0.0125 (10)	0.0013 (9)
C7	0.0760 (14)	0.0398 (13)	0.0702 (13)	0.0018 (10)	0.0174 (11)	0.0001 (9)
C8	0.0681 (12)	0.0434 (14)	0.0686 (13)	−0.0041 (10)	0.0141 (10)	−0.0015 (9)
C9	0.0803 (15)	0.0485 (16)	0.0899 (17)	−0.0003 (12)	0.0024 (13)	0.0028 (12)
C10	0.0638 (13)	0.0487 (15)	0.0766 (14)	−0.0028 (11)	0.0113 (10)	−0.0037 (11)

### Geometric parameters (Å, º)

**Table:**

F1—C4	1.352 (3)	C3—H3	0.9300
N1—C9	1.135 (3)	C4—C5	1.361 (3)
N2—C10	1.136 (3)	C5—C6	1.370 (3)
C1—C6	1.399 (3)	C5—H5	0.9300
C1—C2	1.396 (3)	C6—H6	0.9300
C1—C7	1.443 (3)	C7—C8	1.342 (3)
C2—C3	1.378 (3)	C7—H7	0.9300
C2—H2	0.9300	C8—C10	1.435 (3)
C3—C4	1.372 (3)	C8—C9	1.440 (3)
			
C6—C1—C2	117.5 (2)	C4—C5—H5	120.2
C6—C1—C7	124.74 (19)	C6—C5—H5	120.2
C2—C1—C7	117.8 (2)	C5—C6—C1	120.7 (2)
C3—C2—C1	121.9 (3)	C5—C6—H6	119.6
C3—C2—H2	119.0	C1—C6—H6	119.6
C1—C2—H2	119.0	C8—C7—C1	131.6 (2)
C4—C3—C2	118.0 (2)	C8—C7—H7	114.2
C4—C3—H3	121.0	C1—C7—H7	114.2
C2—C3—H3	121.0	C7—C8—C10	125.6 (2)
F1—C4—C5	119.5 (3)	C7—C8—C9	119.7 (2)
F1—C4—C3	118.2 (2)	C10—C8—C9	114.7 (2)
C5—C4—C3	122.3 (2)	N1—C9—C8	179.3 (3)
C4—C5—C6	119.7 (2)	N2—C10—C8	177.8 (2)
			
C6—C1—C2—C3	−0.2 (3)	C4—C5—C6—C1	0.0 (3)
C7—C1—C2—C3	−178.55 (19)	C2—C1—C6—C5	0.2 (3)
C1—C2—C3—C4	−0.1 (4)	C7—C1—C6—C5	178.50 (19)
C2—C3—C4—F1	−178.78 (19)	C6—C1—C7—C8	1.7 (4)
C2—C3—C4—C5	0.3 (4)	C2—C1—C7—C8	−180.0 (2)
F1—C4—C5—C6	178.84 (19)	C1—C7—C8—C10	0.8 (4)
C3—C4—C5—C6	−0.2 (4)	C1—C7—C8—C9	−179.9 (2)

### Hydrogen-bond geometry (Å, º)

Cg1 is the centroid of the C1–C6 ring.

**Table:**

*D*—H···*A*	*D*—H	H···*A*	*D*···*A*	*D*—H···*A*
C3—H3···N1^i^	0.93	2.61	3.478 (4)	155
C7—H7···N2^ii^	0.93	2.51	3.424 (3)	167
C4—F1···*Cg*1^iii^	1.35 (1)	3.59 (1)	3.573 (2)	79 (1)

Symmetry codes: (i) *x*−1, *y*, *z*−1; (ii) −*x*+2, *y*+1/2, −*z*+3/2; (iii) *x*, −*y*−1/2, *z*−3/2.
